# Efficient Wheat Head Segmentation with Minimal Annotation: A Generative Approach

**DOI:** 10.3390/jimaging10070152

**Published:** 2024-06-21

**Authors:** Jaden Myers, Keyhan Najafian, Farhad Maleki, Katie Ovens

**Affiliations:** 1 Department of Computer Science, University of Calgary, 2500 University Drive NW, Calgary, AB T2N 1N4, Canada; jaden.myers@ucalgary.ca (J.M.); farhad.maleki1@ucalgary.ca (F.M.); 2 Department of Computer Science, University of Saskatchewan, Saskatoon, SK S7N 5A2, Canada; keyhan.najafian@usask.ca

**Keywords:** deep learning, segmentation, generative adversarial networks, data synthesis

## Abstract

Deep learning models have been used for a variety of image processing tasks. However, most of these models are developed through supervised learning approaches, which rely heavily on the availability of large-scale annotated datasets. Developing such datasets is tedious and expensive. In the absence of an annotated dataset, synthetic data can be used for model development; however, due to the substantial differences between simulated and real data, a phenomenon referred to as domain gap, the resulting models often underperform when applied to real data. In this research, we aim to address this challenge by first computationally simulating a large-scale annotated dataset and then using a generative adversarial network (GAN) to fill the gap between simulated and real images. This approach results in a synthetic dataset that can be effectively utilized to train a deep-learning model. Using this approach, we developed a realistic annotated synthetic dataset for wheat head segmentation. This dataset was then used to develop a deep-learning model for semantic segmentation. The resulting model achieved a Dice score of 83.4% on an internal dataset and Dice scores of 79.6% and 83.6% on two external datasets from the Global Wheat Head Detection datasets. While we proposed this approach in the context of wheat head segmentation, it can be generalized to other crop types or, more broadly, to images with dense, repeated patterns such as those found in cellular imagery.

## 1. Introduction

Deep learning models have shown great potential for semantic image segmentation [1,2], where a label is assigned to each pixel representing its semantics. Most semantic segmentation models are developed using supervised approaches that rely on the availability of large amounts of annotated data. However, gathering large amounts of annotated data needed to train these models is often expensive, time-consuming [3], and potentially requires highly specialized expertise to provide the image annotation. The need for a large annotated data set can be alleviated by using synthetic annotated data. However, a model trained on simple synthetic data is unlikely to perform well when applied to real-world data. Therefore, effective integration of synthetic data into segmentation tasks requires overcoming the inherent distribution shift between synthesized and real images, thereby demanding domain adaptation strategies to bridge this gap.

Segmentation has been applied to annotate different plant components to detect abnormalities in crops such as lodging [4], and important phenotypic plant traits in precision agriculture, such as organ size [5], organ health [6], and response to biotic [7] and abiotic stress [8]. Large annotated datasets, such as the Global Wheat Head Detection (GWHD) dataset [9], have enabled the development of new supervised deep learning-based methods for plant phenotyping from field images. Since the pixel-level annotation required for segmentation tasks is substantially more demanding due to the time and effort invested, most of these datasets are focused on classification or object detection tasks and rarely on segmentation tasks.

Some recently proposed solutions for wheat head segmentation aim to accomplish this task with little manual annotation [10,11,12]. In these cases, synthesizing new samples to improve model performance for segmentation tasks is essential, but has the added challenge of distribution shift between the synthesized and real images and requires domain adaptation steps to gradually bridge the domain gap.

Fourati et al. utilized Faster R-CNN and EfficientDet models trained on the GWHD dataset to develop a wheat head detection method [10]. They used semi-supervised techniques such as pseudo-labeling, test time augmentation, multi-scale ensemble, and bootstrap aggregation to improve model performance, and achieved a mean average precision of 0.74. Najafian et al. generated a synthesized dataset using a cut-and-paste approach and trained a YOLO architecture for wheat head detection [11]. Fine-tuning the model with the GWHD dataset resulted in a mean average precision of 0.82. However, their model showed lower performance when trained solely on the synthesized dataset (Precision: 0.318, Recall: 0.130).

Najafian et al. proposed a semi-self-supervised approach for semantic segmentation, utilizing both computationally and manually annotated images. They synthesized an image dataset for semantic segmentation models using a wheat field video clip, a few annotated images, and background scenes [12]. They trained a customized U-Net model with this synthesized dataset and applied domain adaptation steps to address the domain gap between synthesized and real images, and achieved a Dice score of 0.89 and 0.73 on their internal and external test sets, respectively. In this approach, the distinction between the wheat heads and the background in the synthesized images is highly contrasted, making it relatively trivial for a model to learn as it can rely on changes in contrast to perform the segmentation task. Consequently, this method, without further domain adaptation techniques, is not generalizable to wheat head segmentation in real images.

We circumvent these pitfalls by training a model for the segmentation of wheat heads with a modified generative adversarial network (GAN) approach to generate the synthetic data set. A GAN traditionally consists of two subnetworks: a generative network and a discriminative network [13]. The role of the generative network is to generate realistic samples, while the discriminator model takes the generative network’s output as input along with real-world data and provides a binary classification output; true if the input is real or false if it is generated. The parameters of the generative model are adjusted according to the classifications made by the discriminative model and a loss function. The parameters of the discriminative model are adjusted in a similar way based on its classification of the data. The generative and discriminative models will be pitted against each other in a zero-sum game until the discriminative model is no longer able to consistently tell the difference between the synthetic and real data. This process results in a generative network that is able to generate images that closely resemble real-world images.

Cycle-Consistent Generative Adversarial Networks (CycleGANs) are a popular GAN-based deep learning framework focused on the task of unpaired image-to-image translation [14]. For example, they can be used to transform images of a crop field captured under different lighting conditions or with different camera types. This ability to translate images from a source domain to a target domain without the need for paired images allows for data augmentation, domain adaptation, and image enhancement in agriculture applications. The fundamental idea behind CycleGAN is to learn mappings between two different domains without the need for paired training data. Traditional methods for image translation, such as Pix2Pix [15], require paired examples of corresponding images in the source and target domains. However, acquiring such paired data can be challenging and labor-intensive or impractical in many cases. CycleGAN addresses this limitation by using unpaired data for training, making it more flexible and widely applicable. In agriculture, CycleGANs have been utilized for crop disease detection [16], plant phenotyping [17], and crop yield prediction [18].

The limitation of simply using CycleGAN for the purpose of developing segmentation models with accurate annotation is that having an exact match between the generated image and the segmentation itself will not be enforced. A potential consequence of this is that some wheat heads generated do not have a corresponding segmentation, leading to an inaccurate data annotation that will negatively affect downstream models trained on these images. Therefore, we propose to adapt CycleGAN by adding this enforcement step to maintain consistency between generated images and their corresponding annotation.

The paper is structured as follows: In Section 2, we provide an overview of the modified CycleGAN architecture employed to generate synthetic training samples for wheat head segmentation and the process for evaluating the segmentation models. Section 3 presents the results and evaluation of our proposed model. Finally, Section 4 discusses the findings, and Section 5 provides the concluding remarks.

## 2. Materials and Methods

**Figure 1:**
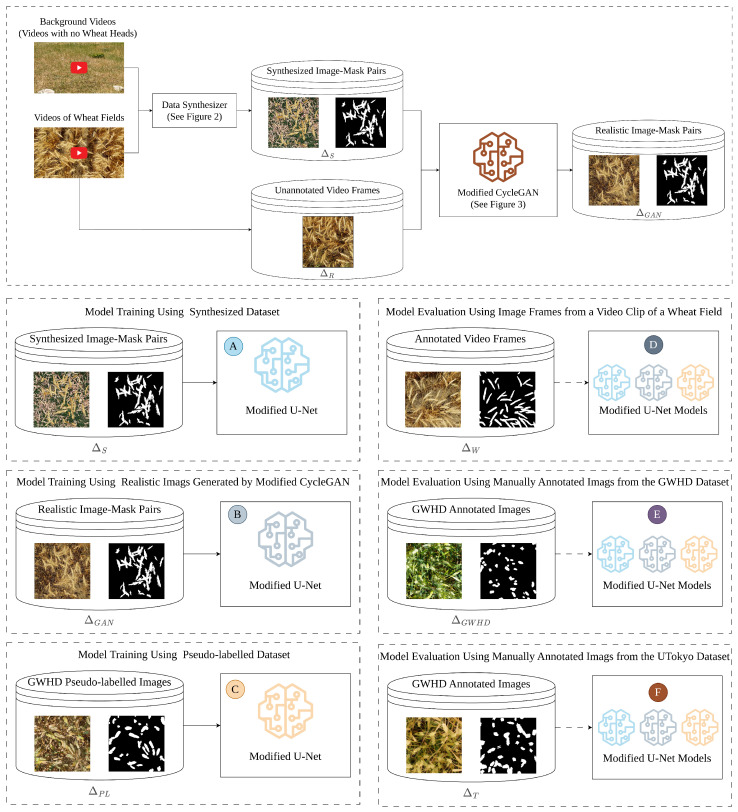
Schematic overview of the model development and evaluation process. The diagram in the top box illustrates a schematic overview of the proposed methodology for data synthesis using the modified CycleGAN model. Annotated images generated from the data synthesizer (see Figure 2) and real unannotated images extracted from video frames are used for development the modified CycleGAN model (see Figure 3). Boxes A, B, and C show the model training process using synthesized computationally annotated images ($\Delta_{S}$), GAN-generated dataset ($\Delta_{GAN}$), and pseudo-labeled dataset ($\Delta_{PL}$), respectively. Boxes D, E, and F show the evaluation process for the three trained models in steps A, B, and C on the internal test set extracted from the video frames ($\Delta_{W}$), the external test set from the GWHD dataset ($\Delta_{GWHD}$), and the external test set from the UTokyo dataset ($\Delta_{Tokyo}$).

This section describes the datasets, the modified CycleGAN for generating synthetic data, and the procedures for training and evaluating the segmentation models (see Figure 1).

### 2.1. Datasets

**Figure 2:**
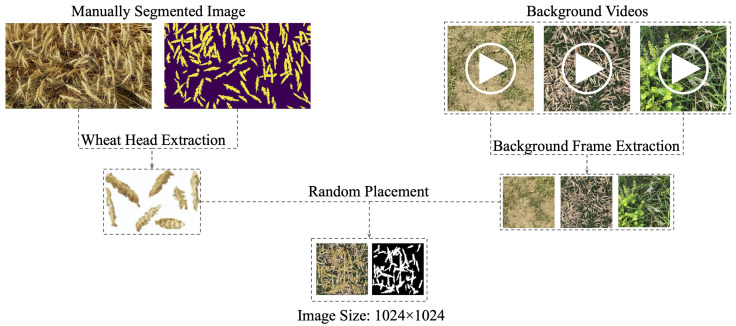
A visualization of the pipeline used to generate synthetic images. Wheat head cutouts are extracted from a manually annotated real wheat image and background frames are extracted from the background videos. The wheat heads are then randomly overlaid onto background frames to generate a wheat head image and its segmentation mask.

The data used in this study can be accessed at https://www.cs.usask.ca/ftp/pub/whs/ (accessed on 1 June 2023). All the data used to train and validate our models came from a video clip of a wheat field, hereafter referred to as *W*, and 11 videos of background scenes captured using Samsung cameras with resolutions of 12 and 48 Megapixels. With only a single frame from *W* that was manually annotated and frames from the background videos, we generated a synthetic dataset $\Delta_{S}$ using the cut-and-paste method proposed by Najafian et al. [12]. Figure 2 illustrates this approach. The dataset $\Delta_{S}$ consists of 11,000 synthetic images and their corresponding semantic segmentation masks. The rest of the frames in *W*, aside from 100 frames used for testing ($\Delta_{W}$), formed the dataset $\Delta_{R}$, consisting of real wheat field images with no semantic segmentation ground truth labels. Our goal is to use the datasets $\Delta_{S}$ and $\Delta_{R}$ to generate a dataset $\Delta_{GAN}$ of GAN-based synthetic images that more closely resemble the real images in $\Delta_{R}$. $\Delta_{GAN}$ consists of 11,000 synthetic images and their corresponding semantic segmentation labels, 10,000 of which were used for training and 1000 for validation of a semantic segmentation model.

To evaluate the performance of the segmentation model trained on our dataset $\Delta_{GAN}$, we tested its performance on 3 different evaluation data sets: (1) an internal dataset $\Delta_{W}$ consisting of the 100 manually annotated images from *W*, (2) an external dataset $\Delta_{GWHD}$ consisting of 365 samples across 18 different domains from the GWHD dataset [9] which was annotated by Najafian et al. [12] to evaluate their model, and (3) another external dataset $\Delta_{Tokyo}$ consisting of samples from the UTokyo subset of the GWHD dataset [9] which was also annotated by Najafian et al. [12] to evaluate their model. These datasets allowed us to compare the performance of our model with the models developed by Najafian et al. [12] and Rawat et al. [19]. Since the internal test set consisting of images from the same video from which we generated the dataset $\Delta_{GAN}$, the performance of the model on the internal test set $\Delta_{W}$ might not be a good indication of the model’s ability to generalize to new domains. However, datasets $\Delta_{GWHD}$ and $\Delta_{Tokyo}$, as external datasets, provide a reliable evaluation of model performance and generalizability. Therefore, we used the two external test datasets—consisting of different varieties of wheat from different fields and at various stages of growth. Samples from these datasets were not seen by the model during the training process.

### 2.2. Model Architecture

Cycle-Consistent Generative Adversarial Network (CycleGAN) architecture was designed for unpaired image-to-image translation [14]. CycleGAN consists of two generator networks and two discriminator networks. One generator transforms images from one domain to another, and the other generator does the reverse. Each generator is paired with a discriminator to distinguish real and fake images in its respective domain. In addition to an adversarial loss function, the CycleGAN model uses a cycle-consistency loss component to minimize the difference between an image and the outcome of translating the same image to another domain and then back to the original domain. By minimizing the combined loss functions, the generator and discriminator networks can learn to perform unsupervised image translation without the need for paired training data.

**Figure 3:**
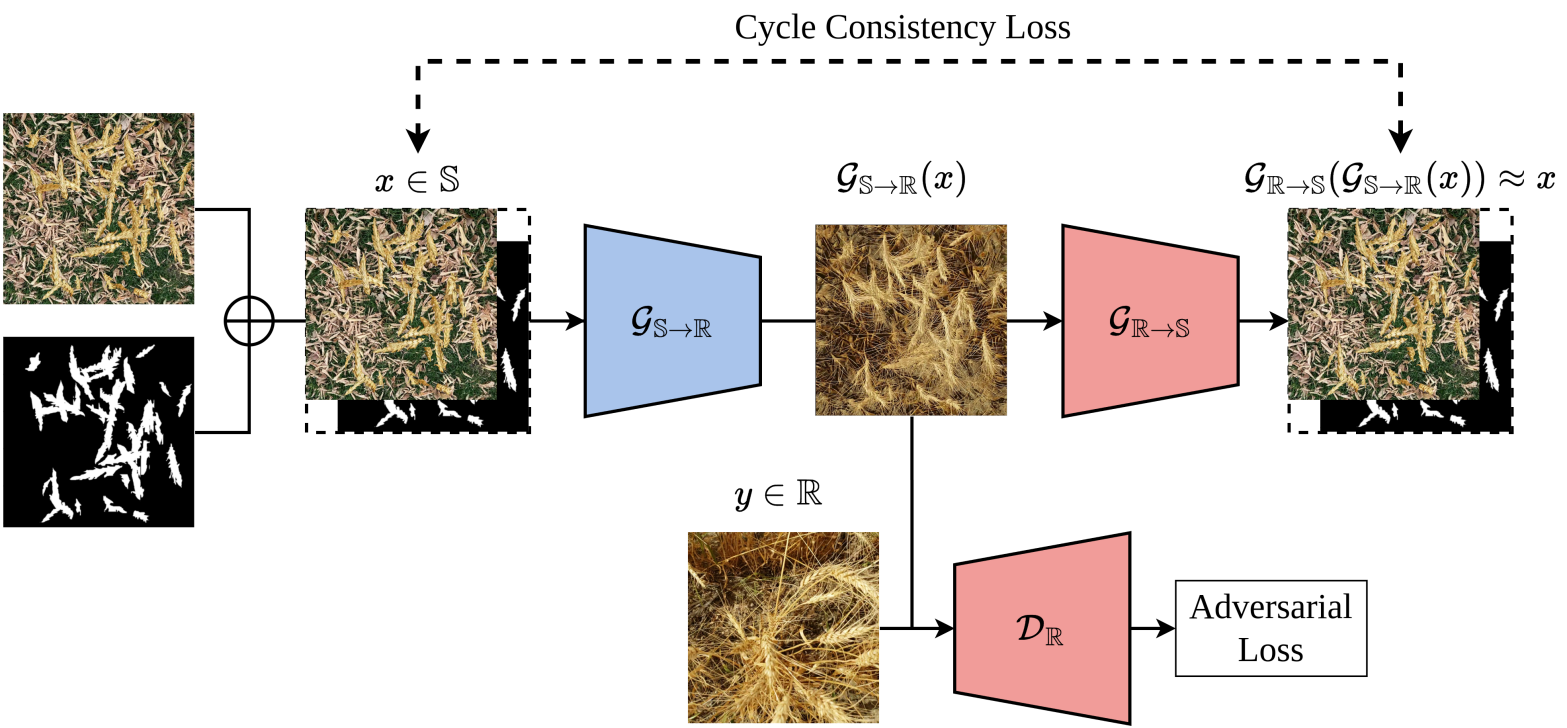
Diagram of the modified CycleGAN. The generator $G_{S\to R}:\;S\to R$ takes as input synthetic images concatenated with their semantic segmentation masks x∈S and outputs a corresponding real image $G_{S\to R}\left(x\right)$. A cycle consistency loss is calculated between *x* and $G_{R\to S}\left(G_{S\to R}\left(x\right)\right)$. Not present in the diagram, a cycle consistency loss is also calculated in the opposite direction with real images y∈R and $G_{S\to R}\left(G_{R\to S}\left(y\right)\right)$, and the discriminator $D_{S}$ calculates an adversarial loss with $G_{R\to S}\left(y\right)\in S$ and *x*.

Although the unmodified CycleGAN focuses on the overall visual appearance, it does not impose any constraints to enforce semantic consistency for the masked regions during the translation process. In this paper, we utilize the copy-paste approach to computationally synthesize images alongside their segmentation mask, and then we develop a CycleGAN-inspired model architecture to transform these synthesized images into realistic images while preserving the annotations. This architecture is presented in Figure 3.

Given two domains S, which represents computationally synthesized images and their segmentation masks, and R, which represents realistic unannotated images, our aim is to transform the synthesized images to make them more realistic, while preserving the segmentation masks, such that the transformed images retain the same segmentation masks as their synthesized counterparts. We utilize a synthesized annotated dataset $\left\{\left(I_{s},M_{s}\right)|I_{s}∼P_{S}\left(I_{s}\right)\;|s=1,\cdots ,n\right\}$—generated based on the methodology proposed by Najafian et al. [12]—from S and a real unannotated dataset $\left\{I_{r}|I_{r}∼P_{R}\left(I_{r}\right)\;r=1,\cdots ,m\right\}$—extracted from video frames of wheat fields—from R. $P_{S}$ and $P_{R}$ represent the probability distribution of data from domains S and R, respectively.

The proposed model architecture consists of two generator networks $G_{S\to R}$ and $G_{R\to S}$ and two discriminator networks $D_{S}$ and $D_{R}$. $G_{S\to R}$ transforms a synthesized image $I_{s}$ from domain S of synthesized images to image $I_{r}$ from domain R of realistic images; the generator $G_{R\to S}$ transforms $I_{r}$ back to an image ${I^{\prime }}_{s}$, which would ideally be the same as $I_{s}$. $D_{S}$ is paired with $G_{R\to S}$, and $D_{R}$ is paired with $G_{S\to R}$ to distinguish real and fake images in their corresponding domains. The CycleGAN model uses a cycle-consistency loss to minimize the difference between $I_{r}$ and ${I^{\prime }}_{r}$. $G_{S\to R}$ is tasked with creating a mapping from domain S to domain R and $G_{R\to S}$ is tasked with creating a mapping from R to S.

$$\begin{array}{cc} G_{S\to R}: & \;\;S\to R\\ G_{R\to S}: & \;\;R\to S \end{array}$$

First, given an input image and its segmentation mask, generator $G_{S\to R}$ produces an image in the style of the target domain R without a segmentation mask. Next, generator $G_{R\to S}$ takes the generated images and tries to regenerate the synthesized input image and its mask from domain S. The cycle consistency loss is then calculated by measuring the distance between the original image and the recreated image, as well as the original and recreated mask. This encourages the model to preserve segmentation masks during domain translations because the masks must be accurately recreated at the end of the cycle.

After generating a realistic and computationally annotated image dataset, we develop an image segmentation model. For the segmentation model, we use the same modified U-Net [20] utilized by Najafian et al. [12]. A binary cross-entropy loss function is used to train all segmentation models. For evaluation of segmentation performance, Dice and IoU are used.

### 2.3. Pseudo Labelling

**Figure 4:**
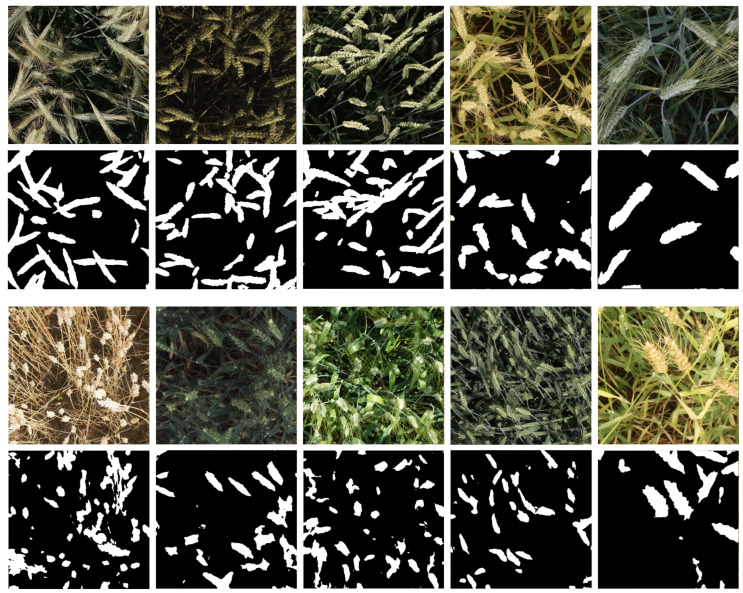
Examples of randomly selected GWHD images and the corresponding pseudo mask predictions. The top row consists of samples that were selected to be part of the dataset used to fine-tune the model. The bottom row consists of samples that were not selected.

To enhance the performance of the segmentation model trained on our GAN-generated dataset, we introduce a domain adaptation step based on the pseudo-labeling approach [21]. From the GWHD dataset, we randomly selected 360 images that are not part of the GWHD external evaluation datasets and passed them as input to the model trained on $\Delta_{GAN}$ to obtain the predicted segmentation masks. From these predictions, we selected only the best predictions, which were then used to fine-tune the model. While highly accurate predictions were chosen manually, the effort needed to choose these predictions by verifying the quality of the predicted segmentation masks is negligible compared to the manual segmentation process. Figure 4 presents examples of images that were selected and not selected. A prediction is considered to be of high quality and should be chosen if it maximizes the area of the wheat head in the image being segmented and minimizes the non-wheat head parts of the image being segmented. A dataset of 99 pseudo-labeled images was compiled using the process.

### 2.4. Experiments

Our model development involves multiple phases which are detailed as follows. We trained our modified CycleGAN model for 60 epochs using the Adam optimizer [22] with a learning rate of 0.0002. We exploited the same optimizer for both pairs of generators and discriminators. We also utilized L1 distance as the cycle loss along with adversarial losses. We used the trained modified CycleGAN model to generate a large-scale computationally annotated dataset with the same size of $\Delta_{S}$, referred to as $\Delta_{GAN}$.

To develop the segmentation model, we closely followed the training strategy utilized by Najafian et al. [12]. We used the same model architecture and trained the model for 45 epochs on the GAN-generated data $\Delta_{GAN}$. In the next phase, using $\Delta_{PL}$ alongside $\Delta_{GAN}$ data, we further fine-tuned the model for 45 more epochs to obtain our final model.

In all three phases of developing our segmentation model, we opted for the SGD optimizer [23] with a learning rate of 0.01, weight decay of 0.001, and momentum of 0.95. We also used Step Scheduler with a reduction rate of 0.1 in every 5 epochs. Moreover, in every step of developing our segmentation model, we evaluated the performance of the model on the validation set using the Dice score as our main score to choose the best-performing model. In each development phase, the best-performing model was evaluated on our test sets. Each model’s performance is detailed and discussed in Section 3 and Section 4.

## 3. Results

### 3.1. Synthesization of Wheat Head Images

**Figure 5:**
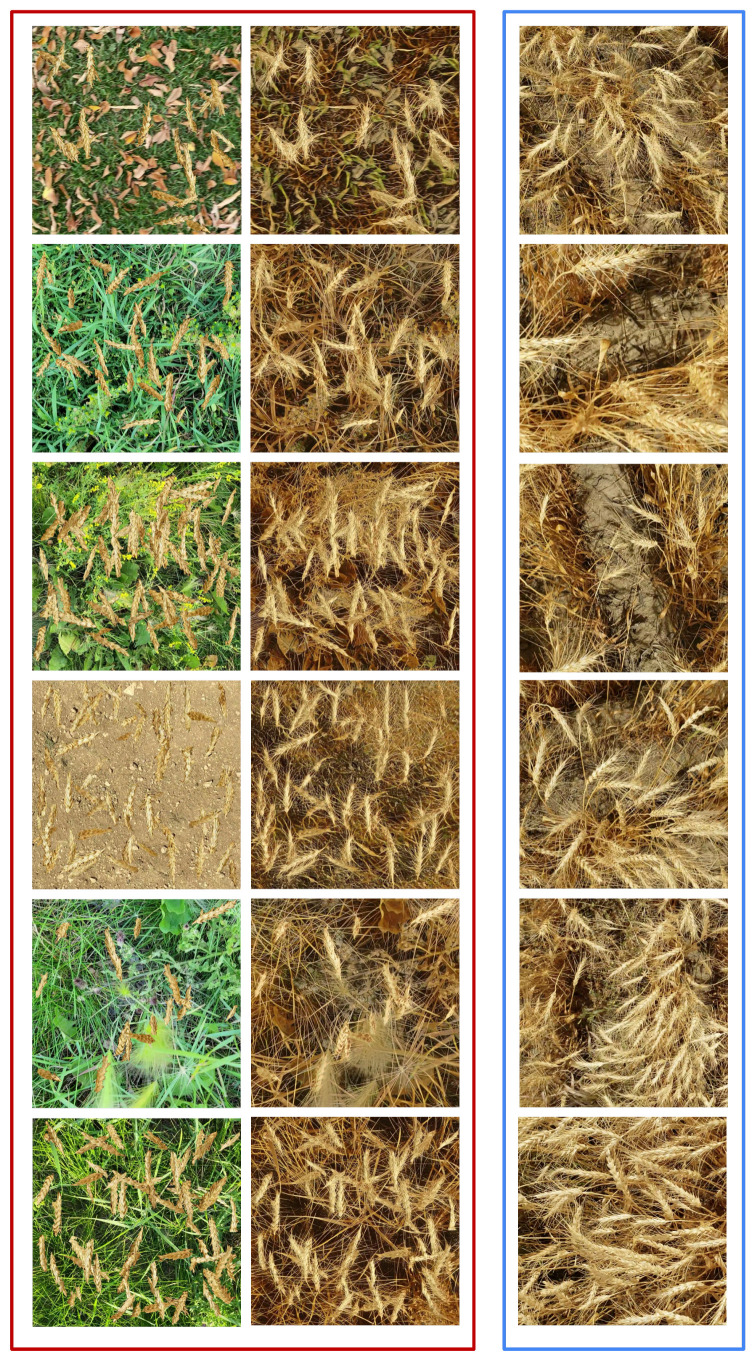
Examples of randomly selected synthetic images from $\Delta_{S}$ (left column) and their corresponding outputs from our modified CycleGAN (right column) are illustrated in the red box. The images in the blue box are randomly selected real wheat field images provided for comparison.

**Figure 6:**
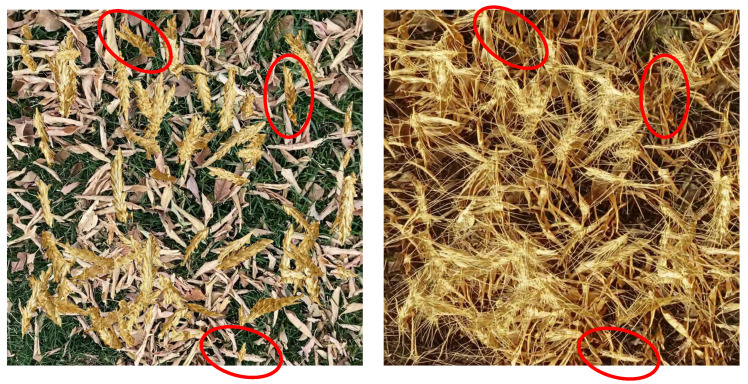
A synthetic wheat image and its corresponding output from an unmodified CycleGAN. The red circles highlight the flaws of the unmodified CycleGAN image translation, where the original CycleGAN does not preserve the same segmentation mask as the input image.

**Figure 7:**
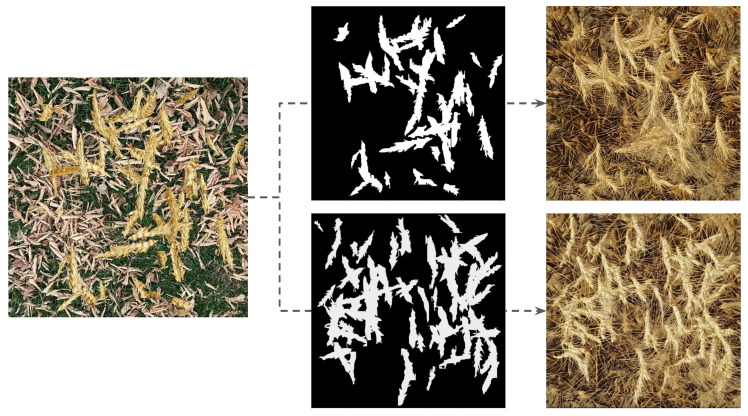
The same synthetic wheat image concatenated with two different semantic segmentation masks and the corresponding output from our modified CycleGAN.

Figure 5 shows randomly selected synthetic images from the dataset $\Delta_{S}$, and their corresponding translated output from the modified CycleGAN in the dataset $\Delta_{GAN}$ with real wheat images for comparison. The translated synthetic images in $\Delta_{GAN}$ are visibly closer to the realistic images compared to the synthetic images from the dataset $\Delta_{S}$. Moreover, although the translated images from the original CycleGAN exhibit equivalent realism to those of our modified CycleGAN, the original CycleGAN does not preserve semantic features during the translation, as illustrated by Figure 6. Our modified CycleGAN with segmentation mask input learned to preserve the semantic features through translation as shown by Figure 7, which illustrates the model’s ability to place wheat heads corresponding to the input segmentation mask.

### 3.2. Evaluation of Wheat Head Segmentation Model Trained with Generated Wheat Head Images

The performance of the segmentation models on both internal and external test sets is outlined in Table 1. Compared to the model trained on the dataset $\Delta_{S}$, the model trained on our modified CycleGAN synthetic dataset $\Delta_{GAN}$ showed a significant increase of 10.2% and 12.0% in the Dice and IoU, respectively, when evaluated on the internal evaluation dataset ($\Delta_{W}$). When evaluated on the external GWHD test set ($\Delta_{GWHD}$) consisting of samples from 18 domains, the performance of the model trained on our dataset showed an even more substantial increase of 21.0% and 16.5% in the Dice and IoU scores, respectively. When evaluated on the UTokyo dataset ($\Delta_{Tokyo}$), the model trained on the data generated from the modified CycleGAN saw an increase of 23.4% and 23.6% in Dice and IoU, respectively. Furthermore, after the model trained on the dataset $\Delta_{GAN}$ was fine-tuned using the pseudo-labeled dataset ($\Delta_{PL}$), the model achieved an even greater increase in performance across all evaluation datasets as shown in Table 1.

The performance ratios between our final model and the baseline model are 1.272, 2.507, and 2.742 for the internal test set ($\Delta_{W}$), GWHD dataset ($\Delta_{GWHD}$), and UTokyo dataset ($\Delta_{Tokyo}$), respectively. These ratios represent the IoU of model C divided by the IoU of model A.

## 4. Discussion

Human-annotated data acquisition is an expensive and labor-intensive task in many domains such as precision agriculture. In this work, we proposed a methodology for computationally generating large-scale labeled training data, for deep learning model development. The proposed approach allowed us to generate a synthetic training dataset, resembling real wheat field images, with little manual annotation. The model trained using this approach resulted in a much smaller domain gap to bridge when applied to real-world data. Our approach demonstrated the potential to be applied to domains, where the lack of annotated data limits the development of deep learning models.

We trained a customized U-Net model [20], utilized by Najafian et al. [12] for the segmentation task in three phases on synthetic, GAN-generated, and a small pseudo-labeled dataset. Comparing the model performance with the state-of-the-art works, our final model obtained a Dice score of 0.796 on $\Delta_{GWHD}$ test set, one of our external sets, while the best model developed by Najafian et al. [12]—trained and fine-tuned on synthetic and pseudo-labeled data—achieved a Dice score of 0.7416 using their proposed Test Time Augmentation (TTA) approach on dataset ($\Delta_{GWHD}$). On the second external test set $\Delta_{Tokyo}$, the best model by Najafian et al. (Model P), which they trained sequentially on the synthetic and a large-scale pseudo-labeled dataset, obtained a Dice score of 0.842. Fine-tuned on a small pseudo-labeled training set of 99 samples, our model obtained a comparable Dice score of 0.836. Moreover, our model surpassed the Rawat et al. [19] model, which was trained in a supervised manner, on the $\Delta_{Tokyo}$. Our approach resulted in an almost 5% increase in IoU score.

### 4.1. Synthetic Image Generation

In the previous state-of-the-art, Najafian et al. [12] used the cut-and-paste method to synthesize computationally annotated images by overlaying extracted wheat heads on the background frames originating from videos with no wheat. Although this approach allows for the generation of large amounts of computationally annotated training data, the resulting data do not closely resemble real data. Consequently, models trained only on such data result in low performance when evaluated on real data. This phenomenon is known as domain/distribution shift. The proposed approach in this research alleviates this problem by adding a new step to the pipeline for image synthesis. We conducted image-to-image translation using a modified CycleGAN to more closely resemble real wheat field images, compared to the simple cut and paste synthetic data. The resulting generated images more closely resemble realistic wheat field images. The model trained on the resulting data, substantially improved performance when compared to initial synthetic data (see Figure 5).

When using a standard/unmodified CycleGAN model, the translated images do not preserve the semantic information, e.g., the location and shape of wheat heads. Consequently, the initial segmentation masks do not match the resulting images. Our modified CycleGAN with semantic mask input was able to generate images that closely resemble real wheat field images while preserving the semantic information of the images. Therefore, the resulting images do not need further manual annotation and the initial segmentation masks can be used along with the resulting image.

### 4.2. Limitations and Future Directions

To reach higher performance, our developed model can be used to predict segmentation masks for real images and the resulting masks often need little-to-no manual curation. This semi-automated approach can expedite the speed in which we develop deep learning models for wheat head segmentation and, in general, crop monitoring, as our approach is generalizable to many other crops. However, minimal manual curation is still needed for such an approach preventing us from fully automating the process. On the other hand, the direct use of pesudo-labelled data, i.e., predictions made by the model, for the further finetuning of the model can introduce noise and systematic bias into the system and is hard to reproduce, which is computationally expensive to fix when the model is trained on non-curated pseudo-labelled images. One potential avenue for future research would be to explore more automated techniques to curate pseudo-labeled data. Techniques such as uncertainty estimation could identify and prioritize the images with the least label uncertainty. Such data paired with computational approaches for image augmentations, to increase data diversity, could allow us to improve model performance without further manual curation.

In our proposed modified CycleGAN, we incorporated the recreation of segmentation masks for the purpose of preserving semantic information during image translation. This is conducted by generating an output which is the concatenation of image and mask. Utilizing a generator component for creating images and another generator model for creating the masks should be explored and the effect of such model architecture on the quality of the resulting images and masks should be studied. Also, a generator architecture such as the U-Net generator proposed by Torbunov et al. [24] could be customized for improving the image generation process. However, we expect a trade-off between the quality of the generated images and semantic preservation.

To train the modified CycleGAN for synthetic-to-real image translation, we used real images extracted from a video clip of a single wheat field. Since all real training images were extracted from the same video clip, the variation within the real training data is low. Utilizing multiple videos taken from multiple wheat fields would increase the variation within the real data and could potentially lead to the data generated by the CycleGAN being more diverse as training data, better representing the variability of real-world data. Incorporating images that bring more variety into the training data, e.g., regarding environmental conditions and growth stage of the wheat fields, would be beneficial for generating realistic images under a variety of different conditions. Having crop images at various stages of development during different growing seasons, weather conditions, and lighting conditions could help to further improve the generalizability of wheat head segmentation models.

In the initial copy–paste image synthesis, wheat heads are randomly overlaid on the background frames. This falls short in precisely emulating the layout of wheat heads in real wheat fields, and our CycleGAN approach tries to preserve this layout. The layout of the initial synthesized wheat field images could be adjusted to more closely resemble that of real images. Creating a pipeline that uses density maps to more closely resemble the rows and orientations of wheat heads in real wheat images, while accounting for variations resulting from wind and movements of crops instead of complete random placement, could further decrease the domain gap between the synthetic and real wheat data.

## 5. Conclusions

In this study, we proposed an approach for generating computationally annotated data to train wheat head segmentation models. Making use of a modified CycleGAN for synthesizing images allowed us to computationally generate large amounts of computationaly annotated images that closely resemble real wheat field images. While we showcased the utility of the proposed approach for wheat head segmentation, the proposed methodology can be applied to other crops and address the challenge of tedious and expensive manual annotation that is often a bottleneck for the full utilization of deep learning approaches for many applications. Our findings highlight the potential of utilizing synthetic data for developing deep learning models in domains where data annotation is tedious or costly.

## Figures and Tables

**Table 1:** The performance of the models trained on the synthetic data sets. Model A was trained on dataset $\Delta_{S}$, which is the synthesized computationally annotated dataset. Model B was trained on dataset $\Delta_{GAN}$, which is generated by our modified CycleGAN model. Model B was fine-tuned using the pseudo-labeled dataset $\Delta_{PL}$ to generate model C.

Model	Trained On	Tested on $\Delta_{W}$		Tested on $\Delta_{\mathrm{GWHD}}$		Tested on $\Delta_{\mathrm{Tokyo}}$	
		Dice	IoU	Dice	IoU	Dice	IoU
A	$\Delta_{S}$	0.709	0.566	0.368	0.274	0.407	0.275
B	$\Delta_{GAN}$	0.811	0.686	0.578	0.440	0.644	0.511
C	$\Delta_{GAN}$ + $\Delta_{PL}$	0.834	0.720	0.796	0.687	0.836	0.754

## Data Availability

Publicly available datasets were utilized in this study. These data can be found here: https://www.cs.usask.ca/ftp/pub/whs/ (accessed on 1 June 2023). The code used to generate synthetic data presented in this study are available on request.

## Abbreviations

The following abbreviations are used in this manuscript:

GAN	Generative Adversarial Network
GWHD	Global Wheat Head Detection
CycleGAN	Cycle-Consistent Generative Adversarial Networks
